# Initial adherence by psychiatric outpatients in a general hospital and relevant personal factors

**DOI:** 10.1186/s12888-022-03797-3

**Published:** 2022-02-21

**Authors:** Minhua Chen, Lina Zhou, Li Ye, Gelin Lin, Yongli Pang, Liyun Lu, Xianglan Wang

**Affiliations:** 1 Department of Psychiatry, Third Affiliated Hospital of Sun Yat-sen University, N0. 600, Tianhe Road, Guangzhou, 510630 China; 2 Department of Psychiatry, Fifth Affiliated Hospital of Sun Yat-sen University, N0. 52, East Meihua Road, Zhuhai, 519000 China

**Keywords:** Psychiatric outpatients, Adherence, Return visit, Pharmacotherapy

## Abstract

**Background:**

Initial adherence is a predictor of long-term adherence and thus is a crucial metric to explore and support. This study aimed to investigate initial adherence by psychiatric outpatients and relevant personal factors.

**Methods:**

The study surveyed psychiatric outpatients using a 30-day timely return visit rate (TRVR) after the first visit to indicate initial adherence. All participants agreed to engage in the self-designed survey and assessments of the Eysenck Personality Questionnaire (EPQ) and Symptoms Checklist-90 (SCL-90). Clients who missed timely return visits received telephone follow-up to determine the main reasons.

**Results:**

The overall TRVR was 59.4, and 40.6% of clients missed return visits. Logistic regression analysis revealed risk factors for initial adherence were work, tense family atmosphere, negative attitudes towards medication, higher EPQ psychoticism score, and lower SCL-90 phobic anxiety score. The main reasons given for non-timely return visits were improvement suggesting lack of need for a return visit, various barriers, no improvement, and side effects.

**Conclusion:**

Psychiatric outpatients had poor initial adherence related to multiple dimensional factors, including job, family, personality characteristics, mental status, and thoughts about mental illness and treatments.

## Background

Adherence is an assessment indicator of a patient’s illness behavior, referring to the extent to which the patient adopts behavior consistent with a treatment plan negotiated by the doctor and patient [1]. The International Society for Pharmacoeconomics and Outcomes Research proposed two types of medical adherence: initial (IMA) and long-term (LTA). IMA refers to adherence after being prescribed a new drug, and LTA refers to adherence during continuous medication or long-term treatment [2, 3]. Poor IMA can predicate worse LTA [4]. Currently, there is no consistent definition of the “initial” phase with most studies defining it as 14 to 28 days after a new prescription or the first visit but others including periods of months or even a year [2]. At the same time, initial adherence is a more widely concept describing illness behavior than IMA. Thus, this study defined the initial phase as 30 days after the first visit and used timely return visit (TRV) behavior as the outcome indicator of initial adherence.

Poor adherence is a global challenge for psychiatric clinical practices [5]. High proportions of psychiatric patients have shown poor treatment adherence, leading to poor treatment outcomes and resulting in a massive waste of social resources [4, 6–10]. Factors found to impact treatment adherence among psychiatric patients are cognitive responses, patients’ trust in doctors, patients’ perception of their mental health, and age, unpleasant side effects of drugs, attitudes towards drugs, paranoia symptoms, substance abuse, self-knowledge, and family environment [11, 12]. There was a rare study on the relationship between personality traits and adherence. A preliminary study showed that psychiatric patients with irritable affective temperament might have poor treatment adherence [13]. However, a pilot study did not find the relationship between personality traits and adherence to antidepressants assessed through self-reporting and electronic monitoring [14].

Most previous studies have focused on long-term medication adherence. The initial adherence in psychiatric outpatients and its relevant factors remain unclear. Thus, the present study investigated the initial adherence in a large sample and performed a telephone follow-up in clients with poor adherence. This study focused on the profile of the initial adherence by psychiatric outpatients and its relevant personal factors. It was assumed that initial adherence was relevant to multiple psychosocial factors, including demographic data, family environment, incoming level, attitudes towards pharmacotherapy, primary diagnosis, symptoms profile, and personality characteristics. Generally, personality traits influence behavioral patterns, and therefore this study assumed they could affect illness behavior such as initial adherence. The telephone survey on reasons for non-timely return visits would much enrich understanding of non-adherence behavior. The findings of this study could provide meaningful evidence for practical efforts to improve initial adherence in psychiatric outpatients.

## Methods

### Participants

All participants were first-visit clients of the psychiatric outpatient department of the Third Affiliated Hospital of Sun Yat-sen University from May to October 2018. The inclusion criteria were as follows: (1) provided informed consent, (2) age between 18 and 65 years, (3) education level above primary school, (4) doctors prescribed medicine at the first visit, and (5) provided complete data. Patients hospitalized after the first visit were excluded. This study was in line with the Declaration of Helsinki, Ethical Principles for Medical Research Involving Human Subjects (World Medical Association, 2013 )[15].

### Survey and Instruments

#### First-visit questionnaire

This self-designed questionnaire was used to investigate age, gender, education level, marital status, employment status, monthly income, residence, family atmosphere, living status, and attitudes towards pharmacotherapy. The respondents completed this survey on their first visit according to their actual circumstances.

#### Timely return visit (TRV)

Based on the hospital information system records, TRV refers to a client completing at least one return visit within 30 days after the first visit; otherwise, the patient was labeled “non-timely return visit” (NTRV). TRV rate (TRVR) = TRV cases number / total sample number × 100%. This study used TRVR as the indicator of initial adherence.

#### Eysenck Personality Questionnaire (EPQ) Chinese version

The EPQ has been widely used to evaluate personality characteristics in China since it was translated into Chinese and standardized in the 1980s [16]. It includes 88 items and four subscales: Psychoticism (P), Extraversion (E), Neuroticism (N), and Lie (L) [17].

#### Symptom checklist 90 (SCL-90) Chinese version

The SCL-90 is usually used to assess mental health [18, 19]. It comprises 90 items and 10 subscales to assess somatization, obsessive-compulsiveness, interpersonal sensitivity, depression, anxiety, hostility, phobic anxiety, paranoid ideation, psychoticism, and additional status (mainly including sleep and eating conditions) [20].

#### Telephone follow-up questionnaire

This questionnaire was self-designed by the researchers. It listed various presupposed reasons for NTRVs: patients’ refusal, unsatisfactory effects, unsatisfactory outpatient environment, improvement leading to believe there is no further need for a return visit, fear of side effects, significant side effects, no time, far distance, failure to make an appointment, economic problems, and others. According to their actual conditions, the respondents could choose one presupposed reason or supply a specific other reason on the telephone.

### Statistic analysis

The dataset was built via SPSS 25.0. Descriptive statistics were used in distributions of the TRVR and reasons for NTRVs. Group comparisons of numerical data such as EPQ and SCL-90 subscale scores were analyzed via an independent t-test. Group comparisons for category data such as age group, marital status, monthly income, and residence were made via *χ*^*2*^ test. The relevant factors for the NTRV were analyzed using Wald forward binary logistic regression. The significance level was set at α = 0.05 except for the significance level of α = 0.1 for selecting independent variables to enroll all potentially relevant factors in the regression process.

## Results

### Investigation into the TRVR and its relevant factors

#### Overall TRVR

In total, 2843 cases meeting inclusion criteria entered statistical analysis, and 1689 completed at least one return visit 30 days after the first visit. The overall TRVR was 59.4%, and 1154 cases (40.6%) missed the TRV (34.2% in patients with psychotic disorders). Thus, participants were divided into two groups: TRV (*n* = 1689) and NTRV (*n* = 1154).

### Demographic data

Table 1 shows the participants’ demographic data and the TRVR distribution among subgroups. There were statistically significant TRVR differences among age, job status, and residence subgroups. Regarding the age groups, the TRVR was highest for the 46–55-year-old subgroup (66.8%) and lowest for the 26–35-year-old subgroup (55.3%) (*χ*^*2*^ *=* 15.409, *P* = 0.004). The TRVR of the on-the-job subgroup was 56.2%, which was significantly higher than that of students or no-job subgroups (*χ*^*2*^ *=* 16.297, *P* < 0.001). The rural residence subgroup had a higher TRVR (63.2%) than the urban residence subgroup (58.2%) (*χ*^*2*^ *=* 10.243, *P* = 0.017).

**Table 1:** General data, attitudes towards pharmacotherapy, primary diagnosis, and distributions of TRVR (N, %)

	N, %	TRV	NTRV	*χ*^*2*^	*P*-value
Gender				0.091	0.763
Male	1165, 41.0	696, 59.7	469, 40.3		
Female	1678, 59.0	993, 59.2	685, 40.8		
Age group				15.409	0.004
18 ~ 25 years	1056, 37.1	630, 59.7	426, 40.3		
26 ~ 35 years	823, 28.9	455, 55.3	368, 44.7		
36 ~ 45 years	473, 16.6	279, 59.0	194, 41.0		
46 ~ 55 years	358, 12.6	239, 66.8	119, 33.2		
56 ~ 65 years	133, 4.7	86, 64.7	47, 35.3		
Educational stage				4.240	0.237
Primary school	158, 5.6	101, 63.9	57, 36.1		
Junior high school	458, 16.1	280, 61.1	178, 38.9		
Senior high school or equivalent	567, 19.9	347, 61.2	220, 38.8		
College and higher	1660, 58.4	961, 57.9	699, 42.1		
Marital status				2.721	0.437
Unmarried	1368, 48.1	816, 59.6	552, 40.4		
Married	1355, 47.7	802, 59.2	553, 40.8		
Divorced	103, 3.6	64, 62.1	39, 37.9		
Widowed	17, 0.6	7, 41.2	10, 58.8		
Work status				16.297	< 0.001
Work	1616, 56.8	908, 56.2	708, 43.8		
Student	562, 19.8	354, 63.0	208, 37.0		
Housework or workless	665, 23.4	427, 64.2	238, 35.8		
Monthly income in RMB				3.900	0.272
< 5 thousands	685, 24.1	412, 60.1	273, 39.9		
5 ~ 10 thousands	1095, 38.5	668, 61.0	427, 39.0		
10 ~ 30 thousands	774, 27.2	449, 58.0	325, 42.0		
> 30 thousands	289, 10.2	160, 55.4	129, 44.6		
Residence				10.243	0.017
Urban	2138, 75.2	1244, 58.2	894, 41.8		
Rural	705, 24.8	445, 63.2	260, 36.9		
Family atmosphere				10.677	0.014
Happy	498, 17.5	323, 64.9	175, 35.1		
Relaxed	900, 31.7	518, 57.6	382, 42.4		
Tense	1189, 41.8	710, 59.7	479, 40.3		
Worse	256, 9.0	138, 53.9	118, 46.1		
Living status				8.962	0.011
With family members	1917, 67.4	1171, 61.1	746, 38.9		
With a lover	165, 5.8	84, 50.9	81, 49.1		
Alone	761, 26.8	434, 57.0	327, 43.0		
Attitudes towards pharmacotherapy				50.807	< 0.001
Willing	387, 13.6	252, 65.1	135, 34.9		
Could accept if necessary	1856, 65.3	1155, 62.2	701, 37.8		
Afraid of side effects	374, 13.2	182, 48.7	192, 51.3		
Refusing	226, 7.9	100, 44.2	126, 55.8		
Primary diagnoses				6.858	0.444
Consultation or observation	239, 8.4	143, 59.8	96, 40.2		
Depression	1257, 44.2	738, 58.7	519, 41.3		
Anxiety	624, 21.9	380, 60.9	244, 39.1		
Bipolar disorder	288, 10.1	178, 61.8	110, 38.2		
Psychotic disorders	73, 2.6	48, 65.8	25, 34.2		
Sleep disorders	141, 5.0	72, 51.1	69, 48.3		
Obsessive-compulsion	54, 1.9	32, 59.3	22, 40.7		
Others	167, 5.9	98, 58.7	69, 41.3		

TRVR: timely return visit rate; TRV: timely return visit; NTRV: non-timely return visit

### Family atmosphere and living status

Table 1 also lists the TRVR distributions related to family atmosphere and living status. Participants with a worse family atmosphere had the lowest TRVR (53.9%), and those with a happy atmosphere had the highest (64.9%) (*χ*^*2*^ *=* 10.677, *P* = 0.014). Participants living with family members had a higher TRVR (61.1%) than those living with a lover or alone (*χ*^*2*^ *=* 8.962, *P* = 0.011).

### Mean EPQ and SCL-90 factor scores

As shown in Table 2, there were no significant differences in mean factor scores of Extraversion, Neuroticism, Psychoticism, and Lie on the EPQ between the TRV and NTRV groups. The TRV group had significantly higher mean factor scores of SCL-90 Somatization (2.05 ± 0.78 vs. 1.99 ± 0.80, *P* = 0.04), Anxiety (2.60 ± 0.93 vs. 2.52 ± 0.95, *P* = 0.026), and Phobic anxiety (2.02 ± 0.84 vs. 1.93 ± 0.84, *P* = 0.009) than the NTRV group.

**Table 2:** Comparisons of EPQ and SCL-90 scores between the timely and non-timely return visit groups (M ± SD)

	TRV	NTRV	*t*	*P*-value
EPQ				
Psychoticism	53.88 ± 10.19	54.53 ± 10.61	−1.662	0.097
Extraversion	47.43 ± 11.04	47.95 ± 11.45	−1.208	0.227
Neuroticism	60.36 ± 11.72	59.73 ± 12.23	1.402	0.639
Lie	46.49 ± 10.44	46.83 ± 10.24	−0.870	0.384
SCL-90				
Somatization	2.05 ± 0.78	1.99 ± 0.80	2.053	0.040
Obsessive-compulsive	2.59 ± 0.88	2.55 ± 0.89	1.302	0.193
Interpersonal sensitivity	2.31 ± 0.92	2.29 ± 0.93	0.477	0.633
Depression	2.68 ± 0.96	2.65 ± 0.98	0.781	0.435
Anxiety	2.60 ± 0.93	2.52 ± 0.95	2.234	0.026
Hostility	2.23 ± 0.91	2.27 ± 0.94	−1.364	0.173
Phobic anxiety	2.02 ± 0.84	1.93 ± 0.84	2.631	0.009
Paranoid ideation	2.07 ± 0.89	2.07 ± 0.89	−0.132	0.895
Psychoticism	2.13 ± 0.80	2.11 ± 0.82	0.628	0.530
Additional items	2.54 ± 0.82	2.48 ± 0.83	1.919	0.055

*TRV* timely return visit, *NTRV* non-timely return visit, *SCL-90* symptoms checklist-90, *EPQ* Eysenck Personality Questionnaire

### Attitudes towards pharmacotherapy and diagnoses

Attitudes towards pharmacotherapy might influence TRVR. Patients willing to receive pharmacotherapy had the highest TRVR (65.1%), and those refusing pharmacotherapy had the lowest (44.2%). The TRVR differences among subgroups of attitudes towards medicine were significant (*χ*^*2*^ = 50.807, *P* < 0.001). Regarding the primary diagnosis, patients with psychotic and bipolar disorder had the two highest TRVRs of 65.8 and 61.8%, respectively, and those with sleep disorders had the lowest (51.1%). However, the TRVR differences among patients with primary diagnoses were not significant (*P* = 0.444). See Table 1.

### Logistic regression model of the NTRV

Let return visit status (0 – TRV, 1 – NTRV) be the dependent variable and significant variables (α = 0.1) related to TRVR in the above statistics such as age groups, working status, residence, family atmosphere, living status, attitudes towards pharmacotherapy, EPQ psychoticism score, and SCL-90 subscale scores of somatization, anxiety, and phobic anxiety be independent variables. The result of the stepwise forward binary logistic regression showed that variables of age groups, working status, family atmosphere, attitudes towards pharmacotherapy, psychoticism score, and phobic anxiety score entered the regression model (*χ*^*2=*^99.060, *df =* 14, *P <* 0.001; overall predicted percentage correct = 61.5%). People aged 46–55 years (*OR* = 0.674, *P* = 0.007, reference to age 18–25 years) with higher SCL-90 phobic anxiety scores (*OR* = 0.851, *P* = 0.001) were at lower risk for NTRV. People with worse (*OR* = 1.540, *P* = 0.008) or relaxed family atmosphere (*OR* = 1.364, *P* = 0.008, reference to happy family atmosphere), attitudes towards pharmacotherapy of refusing medicine (*OR* = 2.265, *P* < 0.001), fear of side effects (*OR* = 1.950, *P* < 0.001, reference to being willing to pharmacotherapy), on the job (*OR* = 1.328, *P* = 0.004, reference to housework or no-job), and higher EPQ psychoticism scores (*OR* = 1.008, *P* = 0.049) were at higher risk for NTRV. See Table 3.

**Table 3:** Logistic regression model of the non-timely return visit

	*B*	*S.E.*	*Wald*	*df*	*P-value*	*Exp(B)*	*95% C.I. for EXP(B)*	
							*Lower*	*Upper*
Age groups			10.792	4	0.029			
18 ~ 25 years			Ref.					
26 ~ 35 years	0.022	0.114	0.038	1	0.846	1.022	0.818	1.278
36 ~ 45 years	−0.126	0.131	0.922	1	0.337	0.882	0.682	1.140
46 ~ 55 years	−0.394	0.146	7.301	1	0.007	0.674	0.506	0.897
56 ~ 65 years	−0.209	0.207	1.025	1	0.311	0.811	0.541	1.217
Family atmosphere			9.452	3	0.024			
Happy			Ref.					
Relax	0.310	0.118	6.935	1	0.008	1.364	1.083	1.719
Tense	0.219	0.115	3.653	1	0.056	1.245	0.994	1.558
Worse	0.432	0.164	6.928	1	0.008	1.540	1.117	2.123
Attitude towards pharmacotherapy			45.289	3	< 0.001			
Willing			Ref.					
Could accept if necessary	0.120	0.119	1.030	1	0.310	1.128	0.894	1.423
Afraid of side effects	0.668	0.152	19.377	1	< 0.001	1.950	1.449	2.626
Refusing pharmacotherapy	0.818	0.174	22.014	1	< 0.001	2.265	1.610	3.187
Working status			13.270	2	0.001			
On the job	0.283	0.098	8.326	1	0.004	1.328	1.095	1.610
Student	−0.074	0.141	0.277	1	0.599	0.928	0.704	1.224
Housework or no job			Ref.					
SCL-90 Phobic anxiety score	−0.162	0.049	10.827	1	0.001	0.851	0.773	0.937
EPQ Psychoticism	0.008	0.004	3.886	1	0.049	1.008	1.000	1.015
Constant	−1.019	0.284	12.918	1	< 0.001	0.361		

*Ref* the reference category of the variable, SCL-90: symptoms checklist-90; EPQ: Eysenck Personality Questionnaire

### Investigation of reasons for NTRV via telephone follow-up

#### The profile of telephone follow-up

Among 1154 clients with NTRVs after the first interview, 693 (60.1%) accepted the telephone investigation, and 461 cases (39.9%) did not. Conditions of follow-up failure included 333 clients (72.2%) that rejected calls, 98 (21.3%) that provided the wrong telephone numbers at the first visit, and 30 (6.5%) that answered the call but refused the investigation.

### Conditions after the first interview

Among 693 cases in which the investigation was completed, 473 (68.4%) clients continued taking medicines according to the prescription (128 of these accepted the advice of return visit as soon as possible, and 346 refused this advice), 73 (10.5%) had continued to visit other hospitals, and 146 (21.1%) had stopped taking medicine and abandoned treatment.

### Self-reported reasons for NTRVs

According to respondents’ answers, the main reasons for NTRVs were as follows: 383 (55.3%) considered the return visit unnecessary because they felt better, 87 (12.6%) felt no improvement, 60 (8.7%) felt noticeable side effects of the medicine, 20 (2.9%) were afraid of side effects of the medicine, 51 (7.4%) reported inconvenience of traveling to the hospital, 61 (8.8%) had no time, 24 (3.5%) failed to make appointments, 4 (0.6%) reported rejecting the return visit, 1 (0.1%) acknowledged difficult economic circumstances, and 2 (0.3%) were unsatisfied with the hospital environment. These reasons were merged and divided into four groups to meet the statistical conditions: improvement and consideration of no need for a return visit (55.3%), no improvement (12.6%), side effects of medicines (11.5%), and various barriers (20.6%). The distribution of self-reported reasons for the NTRV differed significantly among 4 conditions after the first interview (*χ*^*2*^ = 221.989, *P* < 0.001). See Table 4.

**Table 4:** The distribution of reasons for non-timely return visit among conditions after the first interview

Conditions	*N, %*	Self-reported reasons (*N, %*)				*χ*^*2*^	*P-value*
		A (*n* = 87)	B (*n* = 383)	C (*n* = 80)	D (*n* = 143)		
I	128, 18.5	9, 7.0	60, 46.9	11, 8.6	48, 37.5	221.989	< 0.001
II	346, 49.9	28, 8.1	239, 69.1	22, 6.4	57, 16.5		
III	73, 10.5	34, 46.6	7, 9.6	3, 4.1	29, 39.7		
IV	146, 21.1	16, 11.0	77, 52.7	44, 30.1	9, 6.2		

Self-reported reasons: A: No improvement; B: Improvement and belief there is no need for a return visit; C: Medicine side effects; D: Barriers

Conditions: I: Taking medicine and accepting to complete a return visit; II: Taking medicine and unwilling to complete a return visit; III: Transferred to another hospital; IV: Treatment abandonment

### Self-reported reasons for NTRV among varied diagnoses

Table 5 shows self-reported reasons for NTRVs from respondents with different primary diagnoses. Distributions of self-reported reasons for NTRVs of patients with bipolar disorders were similar to that of psychotic disorders, and so as anxiety disorders to obsessive-compulsive disorder (OCD). So we merged data of bipolar disorder and psychotic disorders, anxiety disorders and OCD, respectively, to meet the statistical need. As to reasons for NTRVs, except the diagnosis classification of “others”, patients with sleep disorder (16.3%) and anxiety/OCD (16.1%) reported higher rates of “no improvements” than other diagnosis classifications, especially bipolar disorder/psychotic disorders (8.0%) and consultation or observation (5.7%). Patients with sleep disorder also reported the highest “barriers” rate (32.6%) among all diagnoses. Patients with bipolar disorder/psychotic disorders (13.6%) and depression (12.5%) reported higher rates of “medicine side effects” than other diagnoses, especially sleep disorder (7.0%). However, the *χ*^*2*^ test result showed that the differences above were insignificant (*χ*^*2*^ = 17.829, *P* = 0.272).

**Table 5:** The distribution differences of self-reported reasons for non-timely return visit among primary diagnoses

Diagnoses	*N, %*	Self-reported reasons (*N, %*)				*χ*^*2*^	*P-value*
		A (*n* = 87)	B (*n* = 383)	C (*n* = 80)	D (*n* = 143)		
Consultation or observation	53, 7.6	3, 5.7	33, 62.3	5, 9.4	12, 22.6	17.829	0.272
Depression	305, 44.0	34, 11.1	175, 57.4	38, 12.5	58, 19.0		
Anxiety/OCD	161, 23.2	26, 16.1	85, 52.8	18, 11.2	32, 19.9		
Bipolar disorder/Psychotic disorders	88, 12.7	7, 8.0	51, 58.0	12, 13.6	18, 20.5		
Sleep disorder	43, 6.2	7, 16.3	19, 44.2	3, 7.0	14, 32.6		
others	43, 6.2	10, 23.3	20, 46.5	4, 9.3	9, 20.9		

Self-reported reasons: A: No improvement; B: Improvement and belief there is no need for a return visit; C: Medicine side effects; D: Barriers. OCD: obsessive-compulsive disorder

## Discussion

The present study focused on the initial adherence of psychiatric outpatient first-visit clients in a relatively large sample. We used TRVR as the indicator of the initial adherence and found an overall TRVR of 59.4% as well as its multiple relevant factors, such as attitude towards medication, family atmosphere, working status, age, and EPQ psychoticism and SCL-90 phobic anxiety subscale scores. From the clients’ perspective, through a telephone follow-up, we found that the main reasons for the NTRV included considering return visits unnecessary because of improvement and other various barriers.

Poor initial adherence could decrease the probability of receiving normative and professional treatment, impair outcomes, and even lead to severe consequences [5]. However, poor initial adherence remains a global challenge for psychiatric practices [3, 21]. Ibironke et al. reported a prevalence of 30% of missed first appointments 2–3 weeks after the first outpatient visit in patients with schizophrenia [8]. According to National Comorbidity Survey Replication data, the mental health treatment dropout rate from the general medication sector at 12 months was 31.6%, and over 70% of all dropouts occurred after the first or second visit [22]. A systematic review published in 2021 mentioned that non-adherence ranged between 63 and 74% in patients with schizophrenia and about 50% in patients with bipolar disorder [23]. In Chinese, Yau et al. reported a non-continuous use of antidepressant prevalence of 46% among 189 patients in Hongkong with major depressive disorder newly prescribed antidepressant [24]. Gu et al. investigated the medication adherence within 30 days after the first treatment in 150 first-onset depression outpatients in China mainland and found a poor adherence prevalence of 53.3% (80/150 )[25]. Our findings of high nonadherence prevalence of 40.6% in psychiatric outpatients and 34.2% in patients with psychotic disorders 30 days after the first visit were consistent with the previous studies. They all supported the determination that it is time to pay more attention to and effectively improve initial adherence in psychiatric outpatients.

Generally, initial adherence was among the illness behaviors critically influenced by cognitive appraisal and decision-making by patients based on their perceived information from illness and appropriate treatment acknowledge, feelings related to self-distress, doctor interview, effects, side effects, and balancing benefits against costs [26, 27].

Attitude towards medication markedly influenced initial adherence. People with negative attitudes towards pharmacotherapy had the worst TRVR of 44.2% (Table 1), and refusal of medicine was a key predictor with the highest *OR* of 2.265 to the poor initial adherence (Table 3). At the same time, regarding the self-reported reasons for NTRVs, the present study found that 30.1% of patients abandoned treatments because of medicine side effects (Table 4). In line with the findings of this study, reviews of Kampman [12] and Mitchell [26] also showed that the patient’s choice of treatments was an essential factor influencing adherence. Given this finding and the substantial influence of side effects, we suggest a regular survey before the first visit about attitudes towards treatments, particularly pharmacotherapy, might be constructive for doctors to perform patient education.

Regarding the appraisal of illness severity, patients with higher SCL-90 subscale scores of somatization, anxiety, and phobic anxiety were more likely to complete TRVs (Table 2). The phobic anxiety subscale score was a protective factor for the NTRV (Table 3). A non-adherence study in patients with first-episode psychosis found that less severe positive symptoms at baseline could strongly predict poor medication adherence in the next one and two years [10]. This was similar in patients with depression [7]. These findings suggest that the perception and expectation of significant distress in the body or mind might push patients to carry out suitable initial adherence with less hesitation. Thus, more health education about normative medical intervention should be provided to mild-to-moderate-severity patients and their essential relatives.

This study also represented patients’ balance between benefits and costs of return visits by analyzing the self-reported reasons for NTRVs. For example, patients transferred to other hospitals had higher ratios of “no improvement” (46.6%) and “various barriers” (39.7%) than other subgroups (Table 4). Similarly, being on the job was a risk factor for NTRVs (Table 3), perhaps because a return visit appointment would lead to loss of income. Subsequently, a study on treatment preferences in patients with depression showed that employed patients preferred psychotherapy over antidepressants [28], suggesting that patients on the job might be more afraid of medicine side effects influencing their functional status.

Many factors could influence the decision-making regarding a return visit. Social (including family atmosphere, living status, and residence) and personal factors (including age and personality) were relevant to initial adherence in the present study. A supportive family environment—for example, a happy family atmosphere or living with family members—did help improve initial adherence in this study, which was in line with many previous studies [12, 29, 30]. The influence of age on initial adherence was complex in the present study; there was a tendency of increasing TRVR with age while patients aged 26–35 years old had the worst initial adherence with lower TRVR than those younger or older (Table 1). Consistent with our finding, a structural equation modeling study on adherence to psychopharmacological medications in psychiatric patients reported a weakly positive correlation between adherence and age [11]; in adult outpatients with depression, younger age was related to dropout at 12-week follow-up [30]. Usually, age was a background factor related to various psychological phenomena such as health belief, cognition pattern, social role conflict, and treatment experience and expectation. Therefore, we suspect there might be differing mediating factors between adherence and age in different study participants. Overall, younger adult psychiatric outpatients need more health education about initial adherence.

There were some limitations in this study. As the study was carried out in routine clinical practices, there could be uncontrollable, potentially confounding factors and a relatively high rate of follow-up failure. Although SCL-90 scores could reflect the severity to an extent, this study had not used specific instruments to assess it. Medicines prescribed in the first visit and patients’ reactions might also be essential to the initial adherence, analyzed in another paper for complicated analyses and the length limitation. Additionally, factors related to doctor-patient communications could also influence initial adherence and should be determined in future research. The present study’s findings should be cautiously generalized because of different medical environments among countries and districts. For example, there are apparent differences between healthcare payment policies and accessibility of health resources between China and European countries.

## Conclusions

Poor initial adherence remained in psychiatric outpatients of the general hospital. Health education to patients, particularly younger and mild-severity patients, on the course of illness development, treatment methods, and the necessity of integrated case management are essential for working with psychiatric outpatients.

## Data Availability

The datasets used and analyzed during the current study are available from the corresponding author on reasonable request.

## Abbreviations

IMA: Initial medication adherence
LTA: Long-term medication adherence
TRV: Timely return visit
NTRV: Non-timely return visit
TRVR: Timely return visit rate
EPQ: Eysenck Personality Questionnaire
SCL-90: Symptom checklist 90
